# Sequencing of *Euscaphis konishii* Endocarp Transcriptome Points to Molecular Mechanisms of Endocarp Coloration

**DOI:** 10.3390/ijms19103209

**Published:** 2018-10-17

**Authors:** Xueyan Yuan, Weihong Sun, Xiaoxing Zou, Bobin Liu, Wei Huang, Zeming Chen, Yanlei Li, Meng-Yuan Qiu, Zhong-Jian Liu, Yanling Mao, Shuang-Quan Zou

**Affiliations:** 1 Fujian Colleges and Universities Engineering Research Institute of Conservation and Utilization of Natural Bioresources, College of Forestry, Fujian Agriculture and Forestry University, Fuzhou 350002, China; fafuyxy@163.com (X.Y.); swhjaponica@163.com (W.S.); 000q131012@fafu.edu.cn (X.Z.); liubobin@fafu.edu.cn (B.L.); huangwei@fafu.edu.cn (W.H.); m15705906963@163.com (Z.C.); 15826618747@163.com (Y.L.); fzqmy117@163.com (M.-Y.Q.); 2 Key Laboratory of National Forestry and Grassland Administration for Orchid Conservation and Utilization at Colleage of Landscape Architecture, Fujian Agriculture and Forestry University, Fuzhou 350002, China; zjliu@fafu.edu.cn; 3 Co-Innovation Center for Soil and Water Conservation in Red Soil Region of the Cross-Straits, College of Forestry, Fujian Agriculture and Forestry University, Fuzhou 350002, China; gemubeing@163.com

**Keywords:** endocarp coloration, *Euscaphis konishii*, transcriptome, anthocyanin biosynthesis, chlorophyll degradation

## Abstract

Flower and fruit colors are of vital importance to the ecology and economic market value of plants. The mechanisms of flower and fruit coloration have been well studied, especially among ornamental flower plants and cultivated fruits. As people pay more attention to exocarp coloration, the endocarp coloration in some species has often been ignored. Here, we report on the molecular mechanism of endocarp coloration in three development stages of *Euscaphis konishii*. The results show that endocarp reddening is closely related to anthocyanin accumulation, and a total of 86,120 unigenes were assembled, with a mean length of 893 bp (N50 length of 1642 bp). We identified a large number of differentially expressed genes associated with endocarp coloration, including anthocyanin biosynthesis, carotenoid biosynthesis, and chlorophyll breakdown. The genes participating in each step of the anthocyanin biosynthesis were found in the transcriptome dataset, but a few genes were found in the carotenoid biosynthesis and chlorophyll breakdown. In addition, the candidate R2R3-MYB transcription factors and candidate glutathione S-transferase transport genes, which likely regulate the anthocyanin biosynthesis, were identified. This study offers a platform for *E. konishii* functional genomic research and provides a reference for revealing the regulatory mechanisms of endocarp reddening.

## 1. Introduction

**Figure 1:**
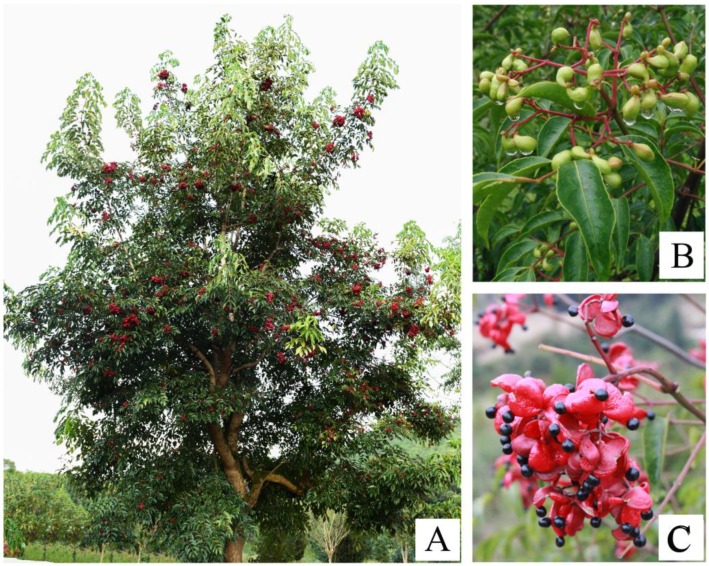
Morphological characteristics of *Euscaphis konishii*: (**A**) fruiting plant, (**B**) young fruits, and (**C**) opening fruits.

In most angiosperms, the flower and fruit colors are not only of vital importance in plant ecology and their ability to attract pollinators, such as insects and birds, and seed-dispersing organisms, but their color is also a crucial trait for both commercial and ornamental value [1,2]. Flower coloration in plants has been well studied, especially among ornamental plants, such as morning glory (*Ipomoea purpurea*), which is the model plant for studying the genetic basis of floricultural traits [3,4,5]; chrysanthemums (*Dendranthema morifolium*) [6,7]; and rose (*Rosa rugosa*) [8]. The fruit color determines the harvest point and increases the market value, and has therefore attracted wide-spread attention resulting in in-depth research, which is focused on the best-selling cultivated fruits, such as apple (*Malus domestica*) [9], grape (*Vitis vinifera*) [10], and strawberry (*Fragaria × ananassa*) [11], for which more attention has been paid to exocarp coloration. However, some species of endocarp coloration have often been ignored. The endocarp is closely related to the seed development, seed protection, and dispersal strategies of seeds [12,13], so studying the molecular mechanisms of endocarp coloration in fruit development may provide a new way to explain the evolutionary and propagation mechanisms. In this study, we examined a tree (*Euscaphis konishii*) that belongs to the Staphyleaceae family, which is distributed across Southeast China, Japan, and Korea [14], that has an endocarp color that changes from green to red during fruit development and the capsule craze, along with a ventral suture, after the fruit turns red (Figure 1). We hypothesize that the reddening endocarp of *E. konishii* is closely associated with the capsule craze and seed dispersal. Sequencing the transcriptome of *E. konishii* pericarp provides a method to reveal the molecular mechanisms of endocarp reddening, and lays the foundation for explaining the capsule craze and seed dispersal mechanisms.

Plant pigments are the key factor affecting fruit coloration, including anthocyanin, carotenoid, and chlorophyll. During fruit development, chlorophyll degradation is accompanied by anthocyanin accumulation or carotenoid retention [15]. Although the mechanisms that control exocarp coloration have been studied in some plant species [16,17,18], the mechanism of pigment catabolism in endocarp coloration is not clear. The change in the *E. konishii* endocarp pigment content during fruit development and maturation has been studied, and the results show that the endocarp color change from green to red is closely related to the anthocyanin accumulation [19]; a comprehensive description of the genes expressed in the *E. konishii* endocarp is lacking. Anthocyanins, a class of flavonoids, are responsible for red, blue, and black plant pigments. Their biosynthetic pathway is well understood, and most of the genes encoding enzymes in this pathway have been isolated and characterized from many plants [20]. To date, approximately six key enzymes (CHS (chalcone synthase), CHI (chalcone isomerase), F3H (flavanone-3-hydroxylase), DFR (dihydroflavonol-4-reductase), ANS (leucoanthocyanidin dioxygenase), and UFGT (UDP (Uridine diphosphate)-flavonoid glucosyltransferase)) are known to be involved in anthocyanin synthesis. A few types of transporters are known to carry anthocyanin from the cytoplasmic surface of the endoplasmic reticulum to the vacuole, such as glutathione S-transferase (GST), multidrug and toxic compound extrusion (MATE), and ATP-binding cassette (ABC) proteins [21]. At least six distinct transcription factors (TFs) (MYB, bHLH, WD40, WRKY, Zinc finger, and MADS box proteins) have been identified in the anthocyanin biosynthesis [22,23].

In general, chlorophyll degradation is obvious during fruit ripening, and fruit coloration is closely related to the content and proportion of chlorophyll [24]. High concentrations of chlorophyll in the pericarp not only mask the red fruit surface color that is provided by other pigments, but also slow their biosynthesis [25,26]. Chlorophyll breakdown is a complex process that involves at least six chlorophyll catabolic enzymes (CCEs), a metal-chelating substance (MCS), and transport mechanisms to deliver chlorophyll breakdown products to the central vacuole [27]. However, chlorophyll degradation in many endocarp species is still poorly understood, and the genes involved in this pathway have not yet been identified in *E. konishii*.

In the present study, we sequenced the transcriptome of the *E. konishii* pericarp in three development stages, and 86,120 unigenes were assembled for the identification of genes corresponding to the pigment metabolic pathways, including chlorophyll degradation, carotenoid biosynthesis, and anthocyanin biosynthesis. This dataset serves as a platform from which to study the regulatory mechanisms of *E. konishii* endocarp reddening.

## 2. Results

### 2.1. Changes in Pigment Content

**Figure 2:**
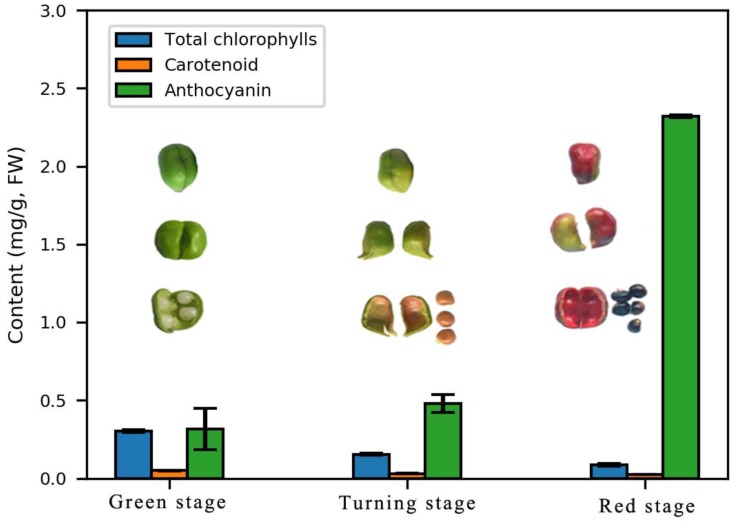
The fruits of *E. konishii* at three developmental stages and contents for the total chlorophyll, carotenoids, and anthocyanins in the pericarp. Green stage: 50 days after flowering; turning stage: 70 days after flowering; red stage: 115 days after flowering; blue bar: total chlorophylls; orange bar: carotenoids; and green bar: anthocyanin.

The endocarp reddening in *E. konishii* may be closely related to the accumulation of the anthocyanin content. During the pericarp development, the total anthocyanin content increased sharply, but the chlorophyll and carotenoid degradation was accompanied by anthocyanin synthesis (Figure 2).

### 2.2. Illumina Sequencing and De Novo Assembly

The library produced 68.78 G of clean data and the average clean data in the nine samples were 6.78 G. RNA-Seq data were used for the statistical analysis so as to ensure the validity of the transcriptome results. In this study, high-quality libraries with mapping rates higher than 79.74% and Q30 values higher than 92.70% were constructed using the pericarp of *E. konishii* in three stages of the endocarp coloration (Table S1). A de novo transcriptome of the pericarp of *E. konishii* was sequenced to study the mechanisms of the endocarp coloration. In total, 86,120 unigenes were assembled, with a mean length of 893 bp (N50 length of 1642 bp) (Table S2), which is similar to the previously reported 894 bp (N50 length of 1307 bp) for *E. konishii* transcriptome in different plant tissues (unpublished; manuscript in preparation), but considerably longer than the 737 bp for the *Litchi chinensis* pericarp transcriptome [28] and 508 bp (N50 length of 635 bp) for the Chinese white pear [29]. These results indicat that the RNA-Seq data of *E. konishii* pericarp were usable in this study.

### 2.3. Functional Annotation of the Unigenes

The gene functions of the 86,120 unigenes were annotated based on eight databases (NR (ftp://ftp.ncbi.nih.gov/blast/db/), Pfam (http://pfam.xfam.org/), GO (http://www.geneontology.org/), KEGG (http://www.genome.jp/kegg/), Swiss-Prot (http://www.uniprot.org/), KOG (ftp://ftp.ncbi.nih.gov/pub/KOG/), eggNOG (http://eggnogdb.embl.de/), and COG (ftp://ftp.ncbi.nih.gov/pub/COG/)) using BLAST comparisons (setting a cut-off E-value of 10^−5^) and HMMER comparisons (setting a cut-off E-value of 10^−10^). The results showed that 39,658 unigenes were significantly similar to known proteins in publicly available databases (Table S3). Approximately 44% of the unigenes were annotated to the NR database, indicating that more than half of the sequences have no apparent homologs, some of which are likely genes with novel functions. Therefore, the transcriptomes of *E. konishii* will serve as an important dataset for the studies of taxa-specific phenomena.

### 2.4. Differentially Expressed Genes between Fruit with Varied Color

**Figure 3:**
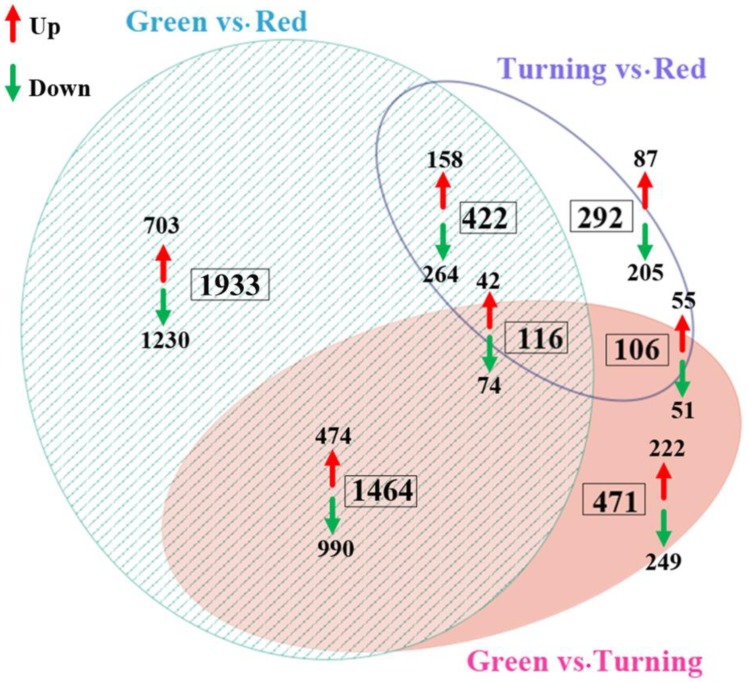
Comparison of differentially expressed genes (DEGs) between any two stages of the *E. konishii* pericarp. The numbers in the boxes indicate the DEGs numbers between any two stages of the *E. konishii* pericarp, and the red and blue arrows represent the numbers of up- and down-regulated genes, respectively.

Based on the three comparisons of green vs. turning, green vs. red, and turning vs. red, we identified a total of 4804 differentially expressed genes (DEGs). Among them, 2175 DEGs between green vs. turning, 3935 DEGs between green vs. red, and 936 DEGs between turning vs. red were detected. In order to identify the related genes that are involved in the regulation of endocarp color, the up- and down-regulated genes between the three stages were further analyzed, and the results showed that the down-regulated genes were more abundant than the up-regulated genes in th special DEGs or co-DEGs of the differential stages (Figure 3).

### 2.5. GO Annotation of DEGs

Among the 4804 DEGs selected to predict functions by gene ontology (GO) annotation, at least one GO term was assigned to the biological processes, cellular components, and molecular functions categories for the 1129 DEGs between green vs. turning, 2107 DEGs between green vs. red, and 543 DEGs between turning vs. red. The three main categories were further classified into 51 functional groups, including 20 biological processes, 16 cellular components, and 15 molecular functions (Figure S1).

### 2.6. Co-Expression Analysis and KEGG Enrichment of DEGs

All of the DEGs were grouped into nine classes by co-expression analysis in this study (Figure S2). An enrichment analysis was performed based on the co-expression results so as to further examine the endocarp coloring gene in *E. konishii*. Four distinct gene expression patterns were enriched (Figure S3) and the results show that the up-regulated genes (Clusters 1 and 2) associated with endocarp color were enriched in anthocyanin biosynthesis, flavonoid biosynthesis, and isoflavonoid biosynthesis. The down-regulated genes (Clusters 3, 4, 5, 7, and 8) were enriched in photosynthesis, photosynthesis-antenna proteins, and anthocyanin. The down first and then up genes (Cluster 6) did not enrich any genes of the endocarp coloration. The up first and then down genes (Cluster 9) were enriched in the photosynthesis-antenna proteins and carotenoid biosynthesis.

### 2.7. Identification of Transcription Factors

Using the online iTAKE 1.6 (http://itak.feilab.net/cgi-bin/itak/online_itak.cgi), and selecting the default parameters to predict the transcriptional factors of 4804 DEGs, (Figure S4 and Table S4), 322 DEGs, representing 6.70% of the total DEG, were annotated to 44 transcription factor (TF) families. Among these TF families, ERF (32) was the most abundant, followed by bHLH (26), MYB_related (26), WRKY (24), MYB (20), NAC (19), bZIP (14), and C2H2 (13).

### 2.8. DEGs Related to Anthocyanin Biosynthesis

**Figure 4:**
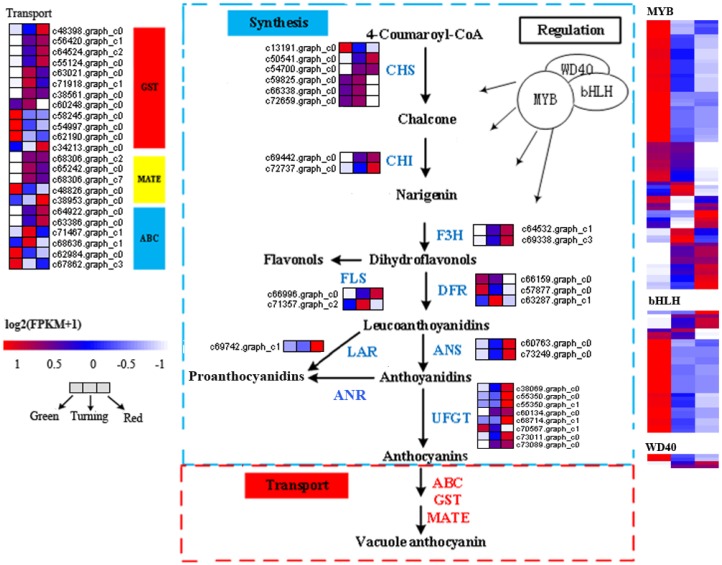
Expression pattern of genes involved in anthocyanin synthesis, regulation, and transport. The value of log2 (FPKM + 1) is represented using the depth of color, with blue representing the up-regulated expression genes and red representing the down-regulated expression genes. FPKM means the fragments per kilobaseof exon per million fragments mapped. CHS—chalcone synthase; CHI—chalcone-flavanone isomerase; F3H—flavanone-3-hydroxylase; DFR—dihydroflavonols 4-reductase; ANS—anthocyanidin synthase; ANR—anthocyanidin reductase; UFGT—UDP (Uridine diphosphate) flavonoid glucosyltransferase; LAR—leucoanthocyanidins reductase; FLS—flavonol synthase; ABC—ATP-binding cassette; GST—glutathione-S-transferase; MATE—multidrug and toxic compound extrusion; MYB—transcription factor MYB; bHLH—basic helix–loop–helix; WD40—WD40 transcription factor.

Twenty- three predicted genes encoding enzymes, including six major enzymes—chalcone synthase (CHS), chalcone isomerase (CHI), flavanone-3-hydroxylase (F3H), dihydroflavonol-4-reductase (DFR), leucoanthocyanidin dioxygenase (ANS), and UDP (Uridine diphosphate)-flavonoid glucosyltransferase (UFGT)—involved in the anthocyanin synthesis were identified (Figure 4 and Table S5). With the exception of DFR, the five types of synthetic anthocyanin genes contained up-regulated genes strongly related to anthocyanin accumulation, including two *CHS* (c50541.graph_c0 and c54700.graph_c0), two *CHI* (c69442.graph_c0 and c72737.graph_c0), two *F3H* (c64532.graph_c1 and c69338.graph_c3), two *ANS* (c60763.graph_c0 and c73249.graph_c0), and seven *UFGT* (c38069.graph_c0, c55350.graph_c0, c55350.graph_c1, c60134.graph_c0, c68714.graph_c1, c73011.graph_c0, and c73089.graph_c0). We also identified one up-regulated flavonol synthase gene (*FLS*, c66996.graph_c0), which may be associated with the *DFR* genes for down-regulation.

The anthocyanin transport was regulated by a series of genes, and 23 predicted anthocyanin transport genes were identified (Figure 4 and Table S5), including 12 GST, 5 MATE, and 6 ABC. Four genes predicted to encode *GST* (c48398.graph_c0, c56420.graph_c1, c64524.graph_c2, and c55124.graph_c0), one *MATE* (c68306.graph_c2), and two *ABC* (c64922.graph_c0 and c63386.graph_c0) were up-regulated in the expression during the fruit development stage. Therefore, these up-regulated genes (four GST, one MATE, and two ABC) were candidate key genes for anthocyanin transport.

We also identified a large number of transcription factors (TF) genes showing a significantly differential expression in the different development stages. A majority of up-regulated or up first and then down TFs, including MYB, hHLH, WD40, WRKY, NAC, ERF, and zinc finger, may regulate anthocyanin biosynthesis [30]. There were 76 TF MYBs, 34 basic helix–loop–helix (bHLH), 4 WD40 TF, 23 WRKY TF, 17 NAC TF, and 32 NAC TF (Figure 4 and Table S5) that changed significantly in expression during the fruit development, but the up-regulated genes were significantly less changed than the down-regulated genes. Only 13 MYBs, 5 bHLHs, 2 WD40, 1 NAC, and 5 ERFs were up-regulated genes and only 8 MYBs, 1 bHLH, and 2 ERFs were up first and then down genes.

### 2.9. Analysis of R2R3-MYB Gene Family

**Figure 5:**
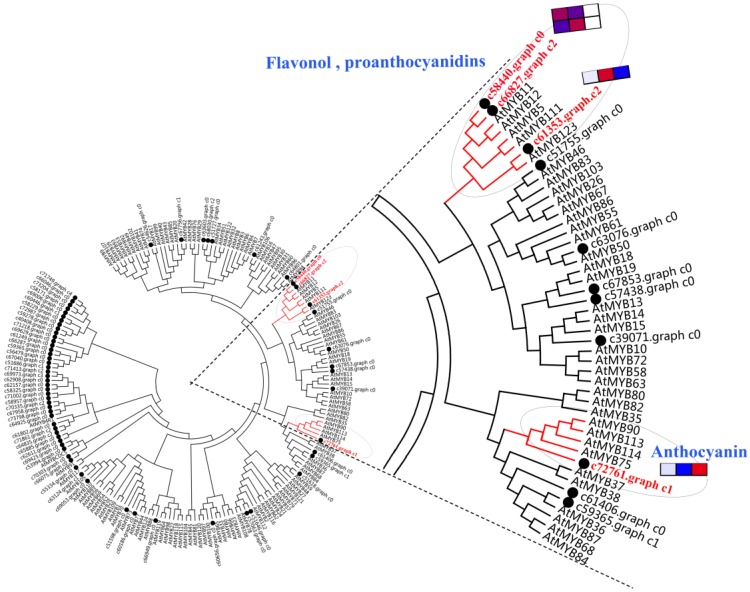
Phylogenetic relationships between *Arabidopsis* R2R3-MYB transcription factors and *E. konishii* MYBs (transcription factor MYB). Black points represent *E. konishii* MYBs and the rest are *Arabidopsis* R2R3-MYB transcription factors.

A phylogenetic tree was constructed using 126 *Arabidopsis thaliana* R2R3-MYB TF family members and candidate *E. konishii* MYB family members (Figure 5). In this phylogenetic tree, two highly homologous *A. thaliana* genes (*AtMYB11* and *AtMYB12*), with the function of controlling the flavonol biosynthesis in all tissues [31], were clustered together with *c58440.graph_c0* and *c66827.graph_c2* genes. Four homologous *A. thaliana* genes (*AtMYB75*, *AtMYB90*, *AtMYB113*, and *AtMYB114*), which regulate the anthocyanin biosynthesis in vegetative tissues [32], were clustered closely together with *c72761.graph_c1*. *AtMYB123*, which controls the biosynthesis of proanthocyanidins in the seed coat of *Arabidopsis*, was clustered together with *c61353.graph_c2* [33].

### 2.10. Protein–Protein Interaction Network Construction and Candidate Gene Selection

**Figure 6:**
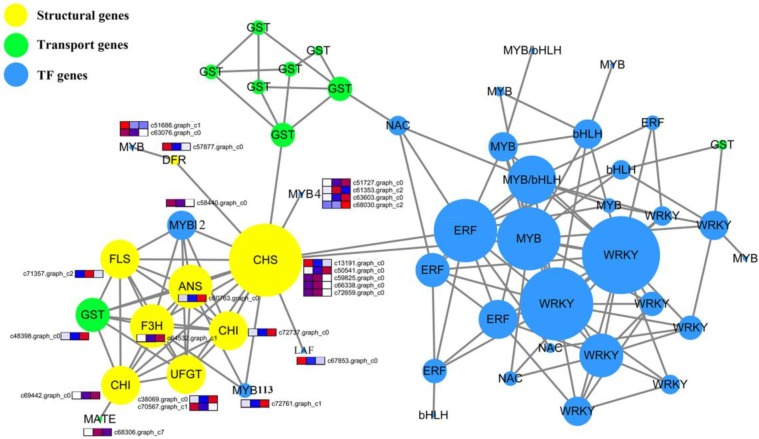
Mapping degree parameter of candidate proteins interaction network to node size. Yellow nodes represent the structure of the genes. Green nodes represent the transport genes. Blue nodes indicate the transcription factor (TF) genes.

A total of 186 transcription factor genes (TFs), 26 synthesis genes, and 23 transport genes may regulate anthocyanin biosynthesis (all genes are shown in Figure 4 and Table S5) in *E. konishii*, and were used to construct a protein interaction network by using the String Online Tools (https://string-db.org/). Then, we constructed a co-expression network by using Cytoscape. The results show that a total of 204 genes were determined between 235 genes, and the study identified three modules from the network (Figure 6). Most of the TF and GST transport genes tended to connect up, respectively. With the exception of DFR, the major synthesis genes (including *CHS (chalcone synthase)*, *CHI (chalcone isomerase)*, *F3H (flavanone-3-hydroxylase)*, *FLS (flavonol synthase)*, *ANS (leucoanthocyanidin dioxygenase)*, and *UFGT (UDP (Uridine diphosphate)-flavonoid glucosyltransferase)*) involved in the anthocyanin biosynthesis closely interacted with each other. One candidate transport gene (*GST*, c48398.graph-c0) and two transcription factors (*MYB12*, c58440.graph-c0; *MYB113*, c72761.graph-c1) strongly interacted with the structure of the genes. The down-regulated *DFR* (c57877.graph_c0) interacted with two down-regulated MYB genes (c51686.graph_c0 and c63076.graph_c0).

### 2.11. Genes Involved in Carotenoid Biosynthesis and Chlorophyll Degradation

Carotenoids are essential components of photosystems that confer a yellow to red coloration to flowers and fruits. In this study, the results showed that only six candidate genes (Table 1), including five down-regulated genes (one *GGPPS (Geranylgeranyl pyrophosphate synthase)*, c64566.graph_c0; one *BOH (beta-carotene hydroxylase)*, c49043.graph_c0; one *ZEP (zeaxanthin epoxidase)*, c68245.graph_c1; and two *NCEDs (9-cis-epoxycarotenoid dioxygenase)*, c64983.graph_c0 and c65617.graph_c0), and one up-regulated gene (*ZEP*, c69135.graph_c0), related to carotenoid biosynthesis, were identified from the DEG of the *E. konishii* pericarp transcriptome database, which was far less than the anthocyanin biosynthesis.

Degreening is obvious during the *E. konishii* fruit ripening, which results from rapid chlorophyll degradation. In this study, five candidates that differentially expressed the genes involved in the chlorophyll breakdown were identified from the *E. konishii* pericarp transcriptome (Table 1), including the genes encoding four chlorophyllase (CLH) and one Mg-dechelatase (MCS). Only one *CLH* (c48268.graph_c0) decreased sharply from the green to the red stage, two *CLH* (c56088.graph_c0 and c69667.graph_c2) were up-regulated genes with low expression in the green stage, but had a high expression in the red stage, and one *CLH* (c66184.graph_c4) decreased sharply in the turning stage, but was high in the red stage. The expression of unigenes c70181.graph_c0 (*MCS*) increased in the turning stage, but decreased in the red stage.

### 2.12. Quantitative Real-Time PCR Validation of Differentially Expressed Transcripts from RNA-Seq

In order to verify the reliability of the RNA-Seq data, nine candidates with differentially expressed transcripts involved in anthocyanin biosynthesis were selected for real-time quantitative polymerase chain reactions (RT-qPCRs) (Table S6). The RT-qPCR results were consistent with those of the RNA-Seq analysis (Figure S5).

## 3. Discussion

### 3.1. Candidate Genes Involved in Anthocyanin Biosynthesis

According to previous studies, the genes of flavonoid biosynthesis in *Arabidopsis* can been divided into “early” genes (such as *CHS*, *CHI*, *F3H*, and *FLS*) and “late” genes (such as *DFR*, *ANS*, *UFGT*, *LAR*, and *ANR*) [23,34]. The transcription levels of the structural genes in the flavonoid biosynthetic pathway are largely regulated by the related transcription factors (such as MYB, hHLH, WD40, WRKY, NAC, ERF, and zinc finger) [30,35]. The early genes (leading to the production of flavonols) and late genes (leading to the production of proanthocyanins and anthocyanins) of the flavonoid biosynthesis pathway in the dicot *Arabidopsis* are activated by coactivator-independent R2R3-MYB transcription factors and the MBW (MYB-bHLH-WD40) complex, respectively [31,32,34]. The anthocyanin accumulation was closely correlated with the coloration of endocarp in *E. konishii* (Figure 2). The genes participating in each step of the anthocyanin biosynthesis pathway were found in the transcriptome dataset of *E. konishii*, and the enzymes were encoded by more than one annotated gene in the anthocyanin biosynthesis (Figure 4 and Table S5). With the exception of DFR, the 17 up-regulated anthocyanin biosynthetic genes (including two *CHS*, two *CHI*, two *F3H*, two *ANS*, and seven *UFGT*) were strongly related to anthocyanin accumulation (Figure 4), which may be determinants of color variation. The up-regulated early genes (*CHS*, *CHI*, *F3H*, and *FLS*) would be regulated by the R2R3-MYB-type gene controlling flavonol biosynthesis (c66827.graph_c2) (Figure 5), to turn a flow of partial production into flavonols, and inhibit the expression of downstream *DFR* genes. In addition, the key regulators of the anthocyanin biosynthesis in *A. thaliana* tissues (*AtMYB75, AtMYB90, AtMYB113, and AtMYB114*) were highly homologous with the MYB tanscription factor (c72761.graph_c1) in *E. konishii* (Figure 5), and the c72761.graph_c1 gene strongly interacted with structure genes (Figure 6). These results suggest that c72761.graph_c1 may be an important regulatory gene influencing the anthocyanin production during the endocarp reddening of *E. konishii.*

In cells, the anthocyanin pigments are synthesized at the cytoplasmic surface of the endoplasmic reticulum and are then transported and finally accumulated inside the vacuole [36]. In the current study, the anthocyanin transport was regulated by a series of enzymes, including GST, ABC, and MATE [21,37,38,39]. GSTs are important anthocyanin transport proteins, as a loss of their function causes a visible deletion of the pigment phenotype in Arabidopsis [40] and Litchi [41]. In this study, four up-regulated GST genes increased sharply during the endocarp ripening and anthocyanin accumulation. A further analysis of the Protein–Protein Interaction(PPI) Network network (Figure 6) showed that c48398.graph-c0 (*GST*) strongly interacted with the structure of genes. These results suggest that c48398.graph-c0 (*GST*) may play a positive role in anthocyanin transport, and may lead to an increase in the vacuolar anthocyanin levels in *E. konishii*. In addition, two major transporter families (MATE and ABC-C) is closely related to the anthocyanin transport in some plants, such as *TT12* from *Arabidopsis* [42] and grape (*Vitis vinifera*) [43]. In the present study, one *MATE* (c68306.graph_c2) and two *ABC* (c64922.graph_c0 and c63386.graph_c0) were the up-regulated genes for fruit coloration. Thus, the MATE and ABC transport genes may be involved in anthocyanin transport.

### 3.2. Candidate Genes Involved in Carotenoid Biosynthesis and Chlorophyll Degradation

Anthocyanins and carotenoids are often present in the same organs, and their combination increases the color variety [20]. However, we found an increase in the anthocyanin content, but a low carotenoid content in *E. konishii*. We also identified six genes in the carotenoid biosynthesis pathway by using transcriptome data (Table 1), including five down-regulated genes and one up-regulated gene. Thus, five down-regulated genes may play a negative role in carotenoid biosynthesis.

Degreening in the senescent leaves and ripening fruit is a natural phenomenon, and research on chlorophyll degradation has been widely reported [44,45]. Chlorophyll is first dephytylated to chlorophyllide by chlorophyllase (CLH), and then a metal-chelating substance (MCS) removes the central Mg atom [27]. In the present study, we only found two up-regulated CLH genes and one up first and then down *MCS (Mg-chelatase)* gene in the *E. konishii* transcriptome database (Table 1). These genes are closely related to chlorophyll breakdown in the reddening endocarp of *E. konishii*.

## 4. Conclusions

The reddening of the *E. konishii* endocarp was closely correlated with anthocyanin accumulation and chlorophyll degradation. The candidate genes involved in the endocarp coloration of *E. konishii* were identified using transcriptome analysis. This study provides a large collection of transcripts and expression profiles associated with *E. konishii* endocarp coloration, including anthocyanin biosynthesis, carotenoid biosynthesis, and chlorophyll breakdown. Most of the genes that we identified participated in the anthocyanin biosynthesis pathway, but a few genes were found for the carotenoid biosynthesis and chlorophyll breakdown. The candidate R2R3-MYB transcription factors that likely regulate anthocyanin biosynthesis were identified, and the candidate GST transport genes involved in the anthocyanin biosynthesis were also identified. This study reveals the regulatory mechanisms of *E. konishii* endocarp reddening and provides a platform for *E. konishii* functional genomic research.

## 5. Material and Methods

### 5.1. Plant Material

The pericarps of *E. konishii* were selected as the plant material and were obtained in 2017 from the nursery stock base of Qingliu County, Fujian Province, China. The pericarps were harvested at three developmental stages of endocarp coloration (green stage, turning stage, and red stage), with three replicates (Figure 2). All of the pericarps from the same stage were collected, frozen immediately in liquid nitrogen, and then stored at −80 °C for further study.

### 5.2. Total Anthocyanin, Carotenoid, and Chlorophyll Determination

The ultrasonic extraction method was used to extract anthocyanin. Approximately 0.5 g of pericarp was collected and quickly ground into powder in liquid nitrogen, before 12 mL of an extract solution (85:15 *v*/*v* mixture of 95% ethanol and 1.5 N HCl) was added, and the sample was extracted for 40 min at 40 °C under a power of 300 W. After filtering, the supernatants were measured for the total anthocyanin content using a pH differential method [46]. The total carotene and chlorophyll were extracted and determined according to the methodology in the Experimental Guidance of Plant Physiology [47]. The experiments were repeated three times.

### 5.3. RNA Extraction and cDNA Synthesis

The total RNA was extracted and purified using an RNAprep Pure Plant Kit (Polysaccharides and Polyphenolics-Rich, Tiangen, Beijing, China), according to the manufacturer’s instructions. The RNA quality was checked using a NanoPhotometer^®^ spectrophotometer (Implen, Westlake Village, CA, USA) and the RNA concentration was measured using Qubit^®^ RNA Assay Kit in a Qubit^®^ 2.0 fluorometer (Life Technologies, Carlsbad, CA, USA). The RNA integrity was assessed using the RNA Nano 6000 Assay Kit, part of the Agilent Bioanalyzer 2100 system (Agilent Technologies, Palo Alto, CA, USA). Firstly, the strand cDNA synthesis was performed using a Revert Aid First Strand cDNA Synthesis Kit (Thermo Fisher, Foster City, CA, USA), according to the manufacturer’s instructions, and was stored at –80 °C for RT-qPCR assays.

### 5.4. Library Construction and Transcriptome Sequencing

The sequencing libraries were generated using NEBNext^®^ Ultra™ RNA Library Prep Kit for Illumina^®^ (San Diego, NE, USA), following the manufacturer’s recommendations, and index codes were added to attribute the sequences to each sample. The library generation involved five steps, as follows: firstly, the mRNA was purified and fragmented; secondly, the double-stranded cDNA was synthesized using the fragmented mRNA; thirdly, the sticky ends of the short fragments were repaired with end repair reagents to avoid self-connection; fourthly, the sequencing adaptors were added to the cDNA fragments that were then enriched by PCR amplification; and finally, the quality control of the constructed libraries was assessed on the Agilent Bioanalyzer 2100 system. The clustering of the index-coded samples was performed on a cBot Cluster Generation System using TruSeq PE Cluster Kit v3-cBot-HS (Illumina, San Diego, NE, USA), according to the manufacturer’s instructions. After the cluster generation, the library preparations were sequenced on an Illumina Hiseq 2000 (San Diego, NE, USA) platform and paired-end reads were generated.

### 5.5. RNA-Sequencing Data Analysis

In order to ensure the accuracy and reliability of the RNA-sequencing data, some of the poor-quality reads were eliminated from the raw reads and only the remaining high-quality reads (clean reads) were used for statistical analysis. The level of gene expression was determined according to the number of fragments per kilobase of exon per million fragments mapped. The genes with a false discovery rate below 0.01 and an absolute value of the log2 (fold change) ≥2 were defined as DEGs. The functional annotation information for these DEGs were obtained using the following databases: NR (NCBI nonredundant protein sequences, ftp://ftp.ncbi.nih.gov/blast/db/), Pfam (Protein family, http://pfam.xfam.org/), KOG/COG/eggNOG (Clusters of Orthologous Groups of proteins, ftp://ftp.ncbi.nih.gov/pub/COG/COG; http://eggnogdb.embl.de/), Swiss-Prot (a manually annotated and reviewed protein sequence database, http://www.uniprot.org/), KEGG (Kyoto Encyclopedia of Genes and Genomes, http://www.genome.jp/kegg/), and GO (Gene Ontology, http://www.geneontology.org/).

### 5.6. RT-qPCR Validation

In order to verify the reliability of the RNA-Seq results, nine important DEGs (the primers used are listed in Table S8) were selected and measured using RT-qPCR on a Quant Studio 5 Real-Time PCR System (Thermo Fisher, Foster City, CA, USA) using the PowerUp^TM^ SYBR^TM^ Green Master Mix (Thermo Fisher, FosterCity, CA, USA), according to the manufacturer’s instructions. UBC23 (ubiquitin-conjugating enzyme 23; the primer used is listed in Table S6) was used as the reference gene, and the relative gene expression levels were determined using the 2^−^^△△CT^ approach [48]. Each sample (including three biological repetitions) was quantified in triplicate. The comparison between the sequencing data and the RT-qPCR results is shown in Figure S5.

### 5.7. Phylogenetic Analysis

The phylogenetic analysis based on the amino acid sequences was performed using MEGA (version 7.0, the laboratory at the Pennsylvania State University, St Collie, PA, USA) and the neighbor joining method with 1000 bootstrap replicates [49]. 

### 5.8. PPI Network Analysis

The network was used to construct a protein interaction network by using the String Online Tools (https://string-db.org/). Then, we constructed a co-expression network using Cytoscape (version 3.6.1, the Institute of Systems Biology, Seattle, Washington, USA) [50].

## Figures and Tables

**Table 1:** Genes involved in carotenoid biosynthesis and chlorophyll degradation.

#ID	Function Annotation	FPKM			Log2 (FC)		
		Green	Turning	Red	G vs. T	G vs. R	T vs. R
**Carotenoid biosynthesis**							
c64566.graph_c0	Geranylgeranyl pyrophosphate synthase (GGPPS)	20.07	6.83	7.35	−1.46	−1.28	0.19
c49043.graph_c0	beta-carotene hydroxylase (BOH)	28.14	9.83	4.58	−1.45	−2.5	−1.04
c68245.graph_c1	zeaxanthin epoxidase (ZEP)	60.19	3.2	4.5	−4.18	−3.62	0.57
c69135.graph_c0	zeaxanthin epoxidase (ZEP)	17.99	27.12	46.58	0.64	1.49	0.86
c64983.graph_c0	9-cis-epoxycarotenoid dioxygenase (NCEDs)	10.53	2.88	1.45	−1.78	−3.22	−1.44
c65617.graph_c0	9-cis-epoxycarotenoid dioxygenase (NCEDs)	49.88	16.86	8.44	−1.5	−2.44	−0.94
**Chlorophyll degradation**							
c48268.graph_c0	Chlorophyllase (CLH)	43.88	14.36	51.63	−1.46	0.45	1.92
c56088.graph_c0	Chlorophyllase (CLH)	16.83	73.89	114.68	2.18	2.87	0.71
c66184.graph_c4	Chlorophyllase (CLH)	2.24	0	0	/	/	/
c69667.graph_c2	Chlorophyllase (CLH)	26.13	33.15	70.04	0.34	1.38	1.06
c70181.graph_c0	Mg-chelatase (MCS)	6.77	17.3	15.64	1.44	1.34	−0.08

G—green stage; T—turning stage; R—red stage; FPKM—fragments per kilobaseof exon per million fragments mapped.

## Supplementary Materials

Supplementary materials can be found at http://www.mdpi.com/1422-0067/19/10/3209/s1.
