# Supplementary material for: Sequencing of Euscaphis konishii Endocarp Transcriptome Points to Molecular Mechanisms of Endocarp Coloration
Source: Int J Mol Sci. 2018 Oct 17;19(10):3209. doi: 10.3390/ijms19103209 (PMC6214000; doi:10.3390/ijms19103209)
Supplement: Supplementary file 1 [file ijms-19-03209-s001.zip › Supplementary Figures.pdf]

Supplementary Figures

Sequencing of *Euscaphis konishii* Endocarp Transcriptome Points to Molecular Mechanisms of Endocarp Coloration

Xueyan Yuan [1,2], Weihong Sun [1,2], Xiaoxing Zou [1], Bobin Liu [1], Wei Huang [1], Zeming Chen [1], Yanlei Li [1], Meng-Yuan Qiu [1], Zhong-Jian Liu [2], Yanling Mao [3] and Shuang-Quan Zou [1,2,3,*]

[1] Fujian Colleges and Universities Engineering Research Institute of Conservation and Utilization of Natural Bioresources, College of Forestry, Fujian Agriculture and Forestry University, Fuzhou 350002, China; fafuyxy@163.com (X.Y.); swhjaponica@163.com (W.S.); 000q131012@fafu.edu.cn (X.Z.); liubobin@fafu.edu.cn (B.L.); huangwei@fafu.edu.cn (W.H.); m15705906963@163.com (Z.C.); 15826618747@163.com (Y.L.); fzqmy117@163.com (M.-Y.Q.); gemubeing@163.com (Y. M.)

[2] Key Laboratory of National Forestry and Grassland Administration for Orchid Conservation and Utilization at Colleage of Landscape Architecture, Fujian Agriculture and Forestry University, Fuzhou 350002, China; zjliu@fafu.edu.cn(Z.-J. L.)

[3] Co-Innovation Center for Soil and Water Conservation in Red Soil Region of the Cross-Straits, College of Forestry, Fujian Agriculture and Forestry University, Fuzhou 350002, China;

* Correspondence: zou@fafu.edu.cn; Tel.: +86-591-8375-5026

Figure legend

Supplementary Figure 1. GO annotation of DEGs.

Supplementary Figure 2. Co expression analysis of DEGs.

Supplementary Figure 3. KEGG enrichment of DEGs.

Supplementary Figure 4. DEGs distribution of TFs in different fruit development stages.

Supplementary Figure 5. Comparison of sequencing data and qRT-PCR results. Error bars indicate the standard deviation (SD) of means (n = 3).

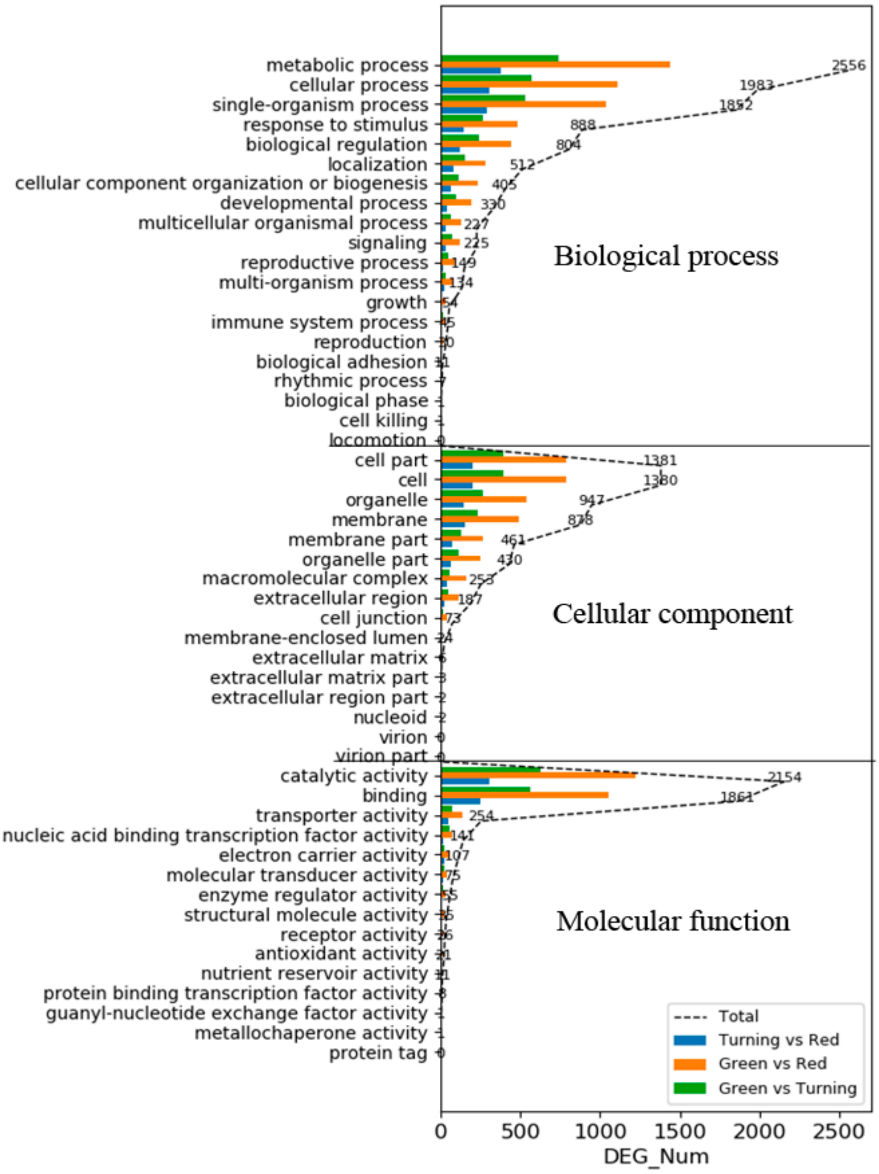

Supplementary Figure 1. GO annotation of DEGs

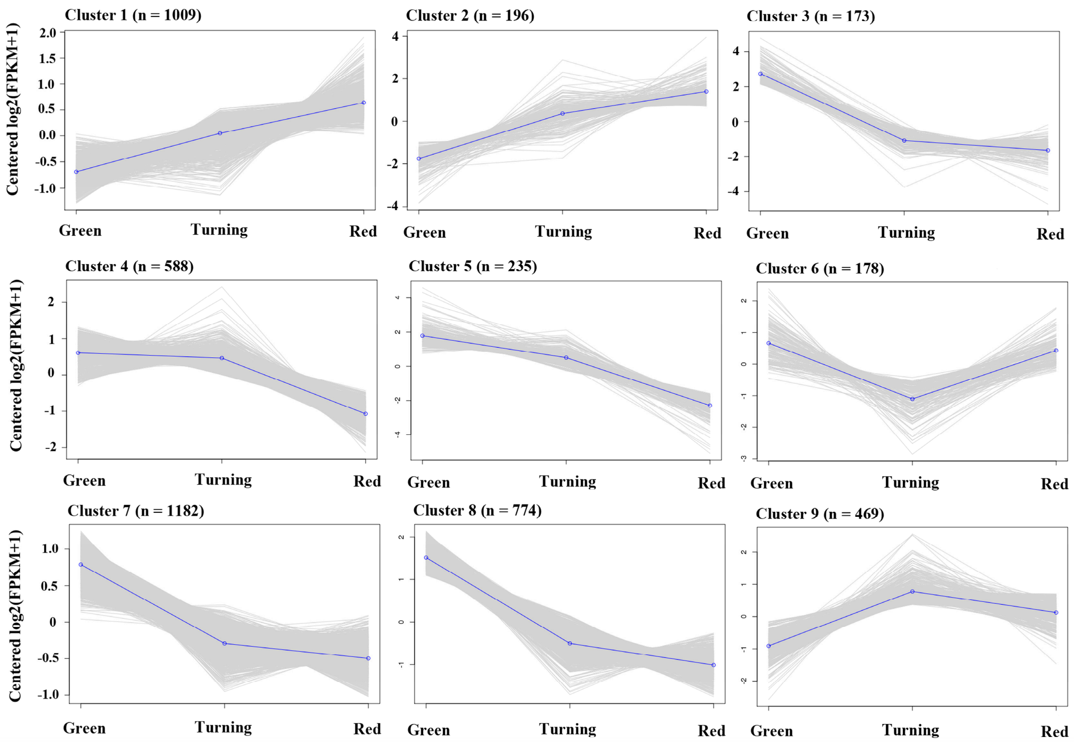

Supplementary Figure 2. Co expression analysis of DEGs

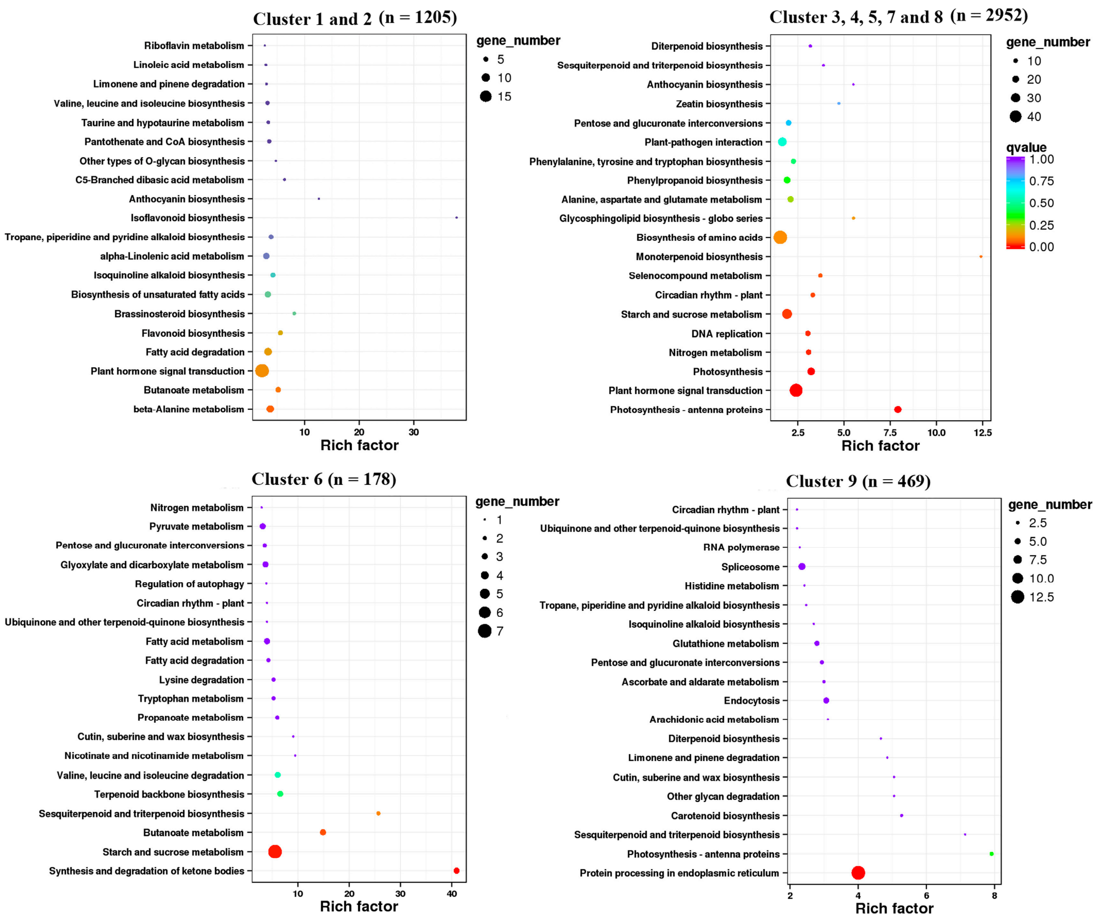

Supplementary Figure 3. KEGG enrichment of DEGs

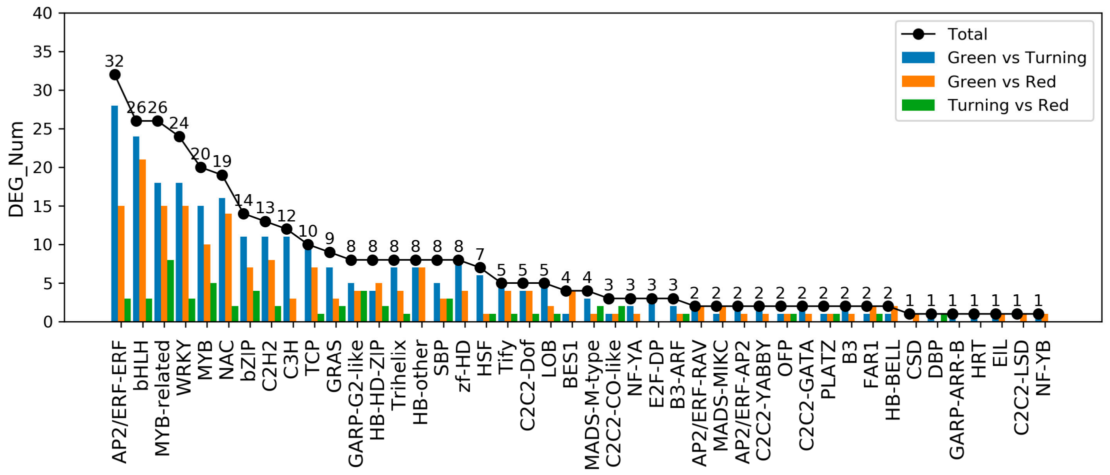

Supplementary Figure 4. DEGs distribution of TFs in different fruit development stages

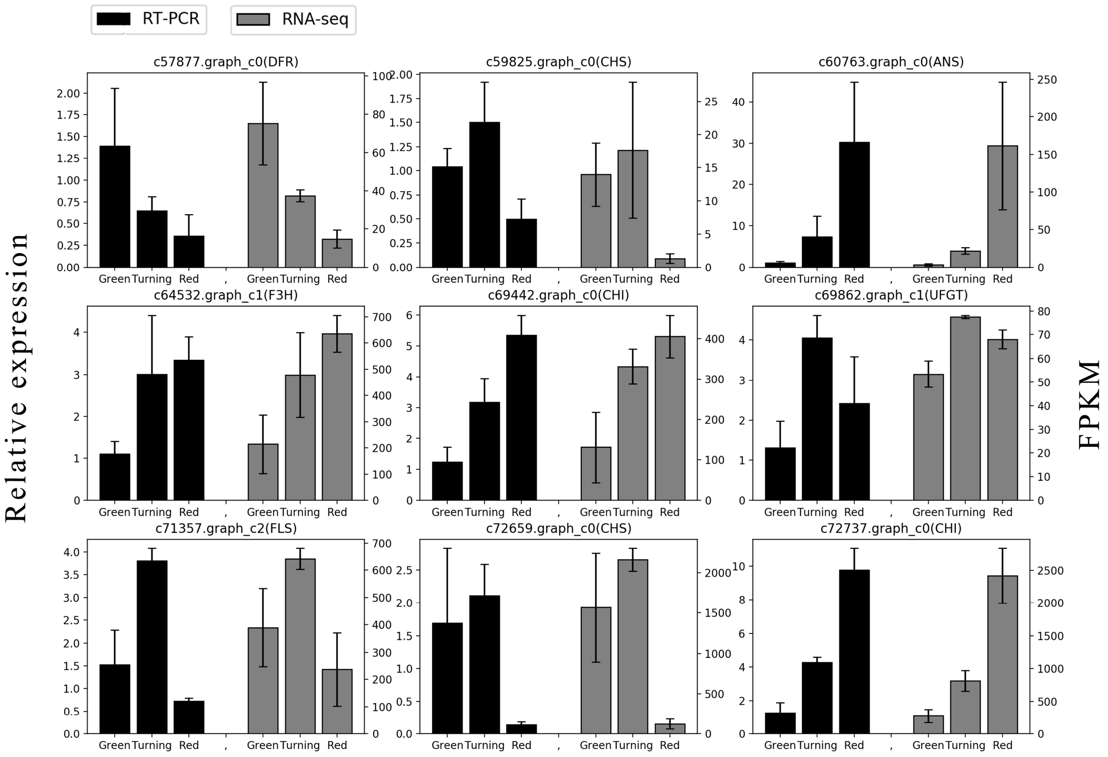

Supplementary Figure 5. Comparison of sequencing data and qRT-PCR results. Error bars indicate the standard deviation (SD) of means (n = 3).
